# Supplementary material for: Physical networks from entropy-driven non-covalent interactions
Source: Nat Commun. 2021 Feb 2;12:746. doi: 10.1038/s41467-021-21024-7 (PMC7854746; doi:10.1038/s41467-021-21024-7)
Supplement: Supplementary file 1 — Supplementary Information [file 41467_2021_21024_MOESM1_ESM.pdf]

Supplementary Information

Physical networks from entropy-driven non-covalent interactions

Anthony C. Yu,[1] Huada Lian,[1,2] Xian Kong,[2] Hector Lopez Hernandez,[1] Jian Qin,[2] and Eric A. Appel[1]*

[1] Department of Materials Science & Engineering, Stanford University, Stanford CA 94305

[2] Department of Chemical Engineering, Stanford University, Stanford CA 94305

*Person to whom correspondence should be addressed:
Dr. Eric A. Appel, eappel@stanford.edu

Supplementary Tables

Supplementary Table 1. Thermodynamic parameters determined by ITC experiments.

	ΔG° (kJ/mol)	ΔH° (kJ/mol)	$T\Delta S^{\circ}$ (kJ/mol)	K_{eq}
PSNP/HPMC-C_{12}	-18.3	23.8	42.5	1.72×10^{3}
CD/Ad[1]	-23.8	-32.2	-8.40	1.49×10^{4}
CB[8]/Np[2]	-28.1	-55.7	-27.6	8.43×10^{4}
Ca/Alginate[3]	-23.0	-15.0	7.99	1.08×10^{4}

Supplementary Figures

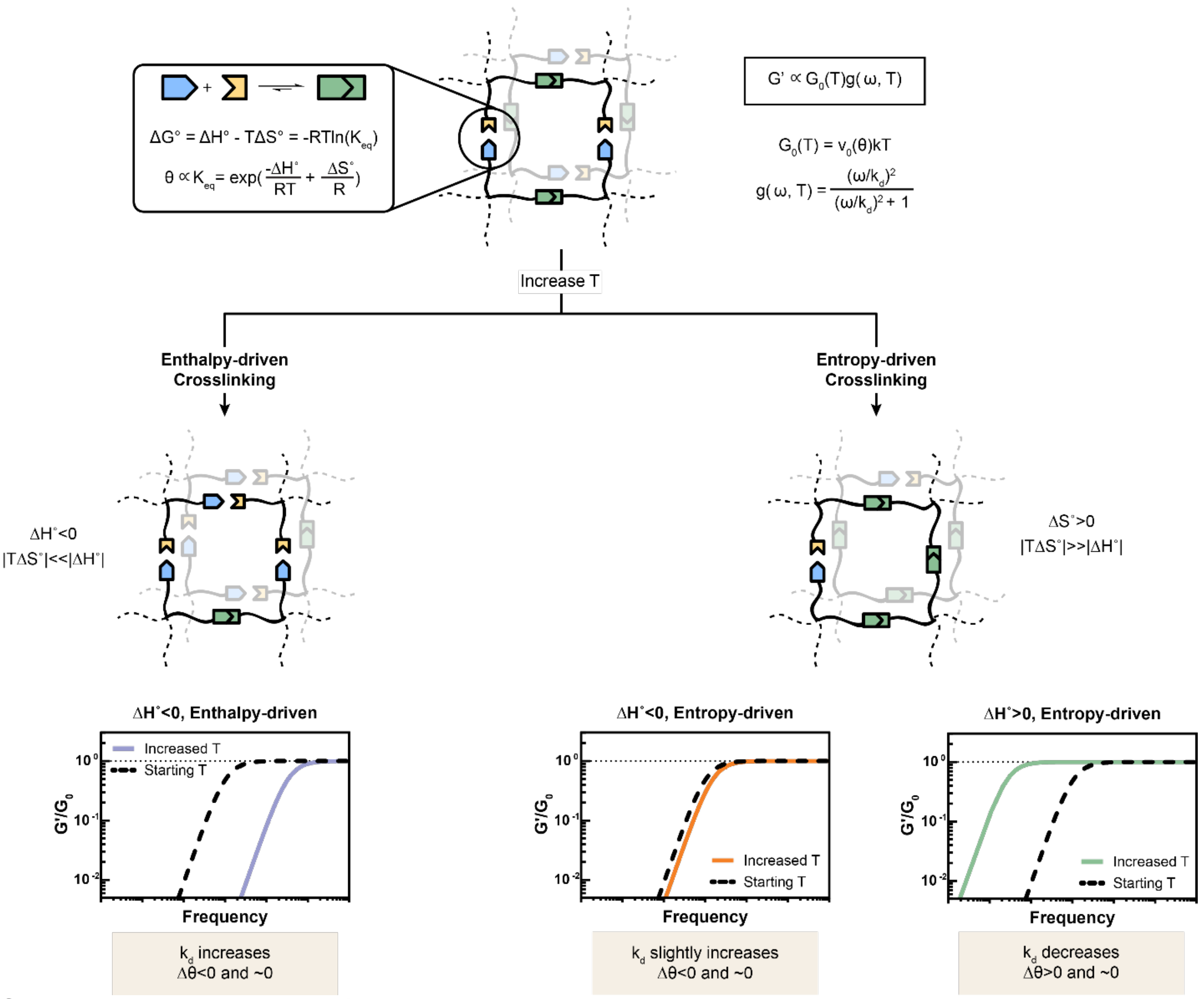

Supplementary Figure 1. Network dependence on crosslinking thermodynamics. Schematic illustrating enthalpy-driven versus entropy-driven physical crosslinking interactions. Enthalpy-driven interactions exhibit increased dissociation rate constants (k_d) and negligible decreases in the proportion of bound crosslinks (θ). The resulting normalized shear modulus (G'/G_0) shifts to the right towards higher frequencies at elevated temperatures. Entropy-driven interactions can be separated into two classes: exothermic or endothermic. For exothermic, entropy-driven crosslinks, k_d exhibits a small increase that is dampened by the large entropy contribution, resulting in a negligible horizontal shift in the normalized modulus. For endothermic, entropy-driven crosslinks, k_d decreases and the normalized modulus shifts to the left at elevated temperatures.

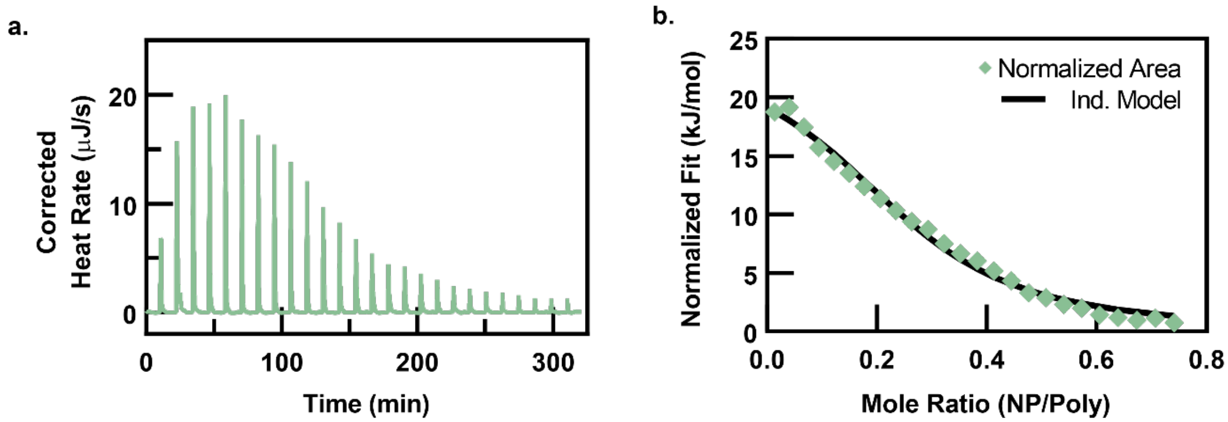

Supplementary Figure 2. Isothermal titration calorimetry (ITC) data. a, Raw heat data for PSNP/HPMC-C_{12} solutions using incremental injections of PSNPs into a HPMC-C_{12} solution. **b,** Normalized integrated heats of the incremental ITC experiment show positive values, indicating an endothermic interaction.

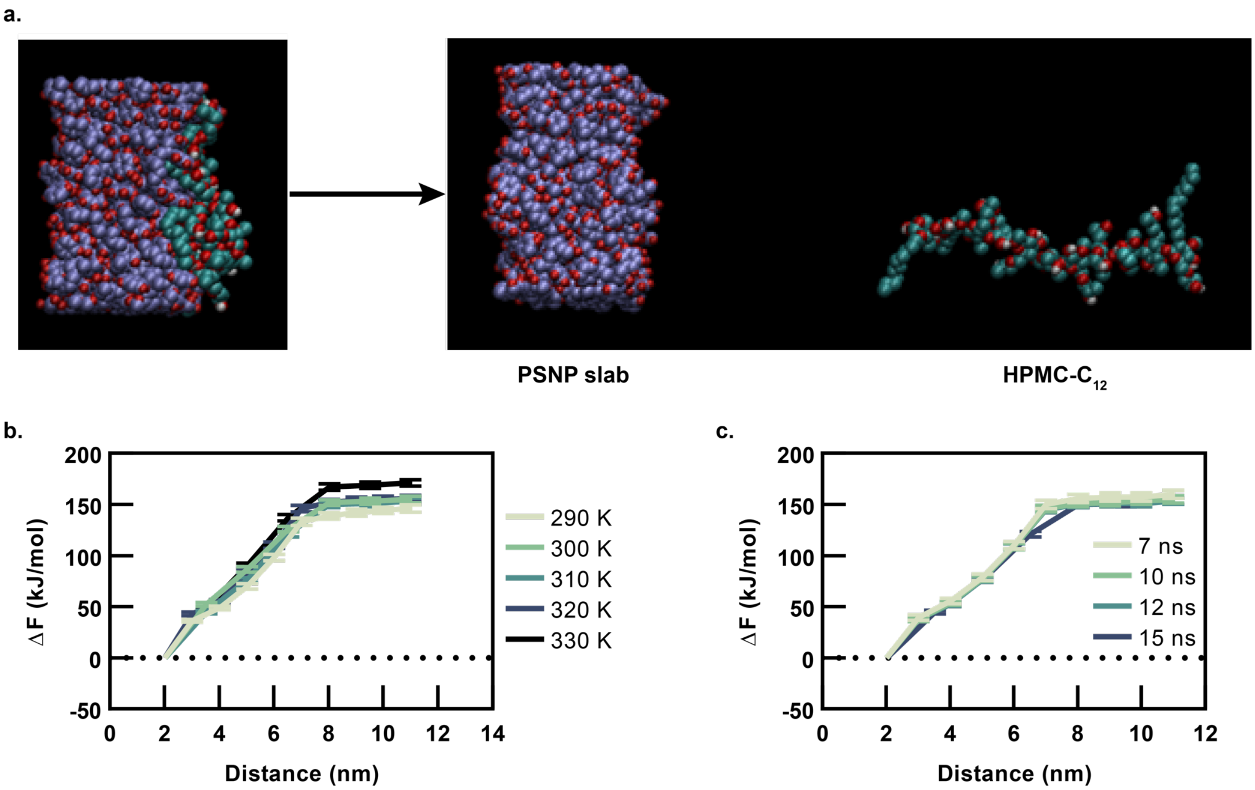

Supplementary Figure 3. Simulation data. a, Simulation screenshots of equilibrated PSNP and HPMC-C_{12} at its binding state and one initial configuration of a window of the umbrella sampling. **b,** Plot that illustrates the free energy vs distance plot for each temperature. **c,** Convergence of PMF constructed by the last 7, 10, 12, and 15 ns samples at 310 K.

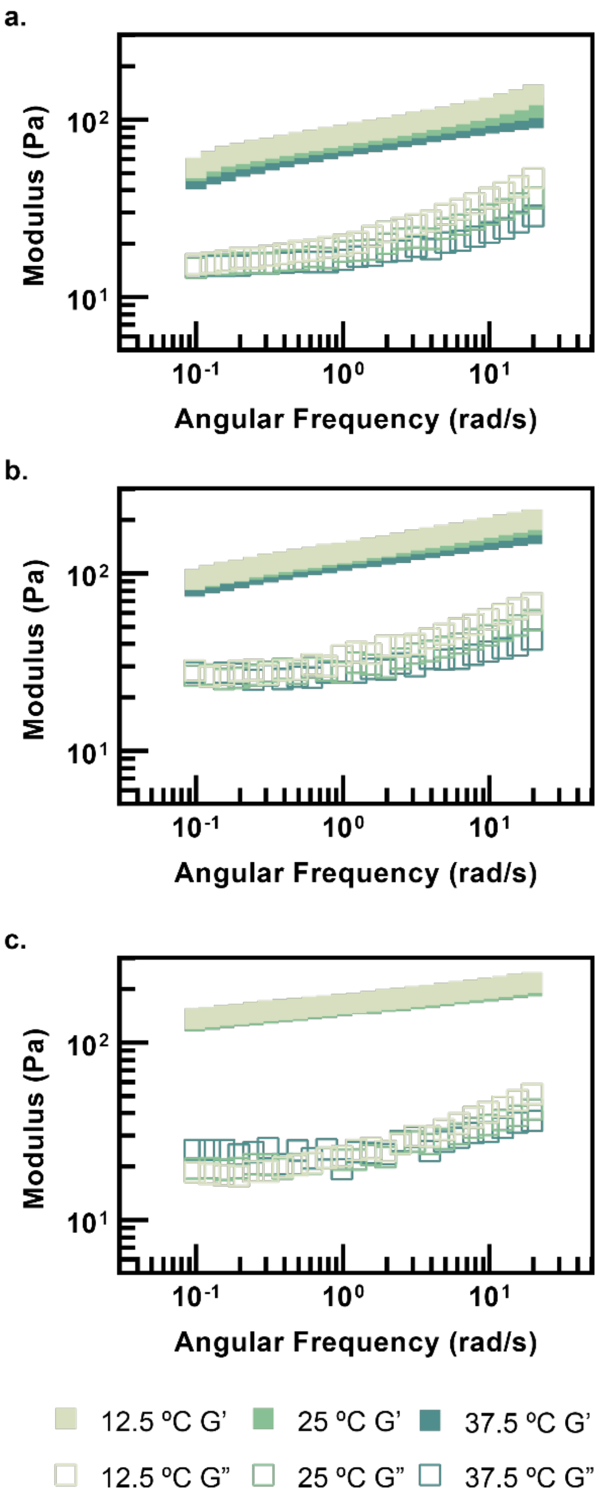

Supplementary Figure 4. Frequency sweep replicates with different temperature conditioning. a, Data plotted in the main text. Each curve was measured from one sample aliquot per temperature. **b,** In this case the sample was loaded at 12.5°C and measurements were taken from low to high temperatures, allowing the material to equilibrate at each temperature. **c,** The sample was loaded at 37°C and measurements were taken from high to low temperatures, allowing the material to equilibrate at each temperature.

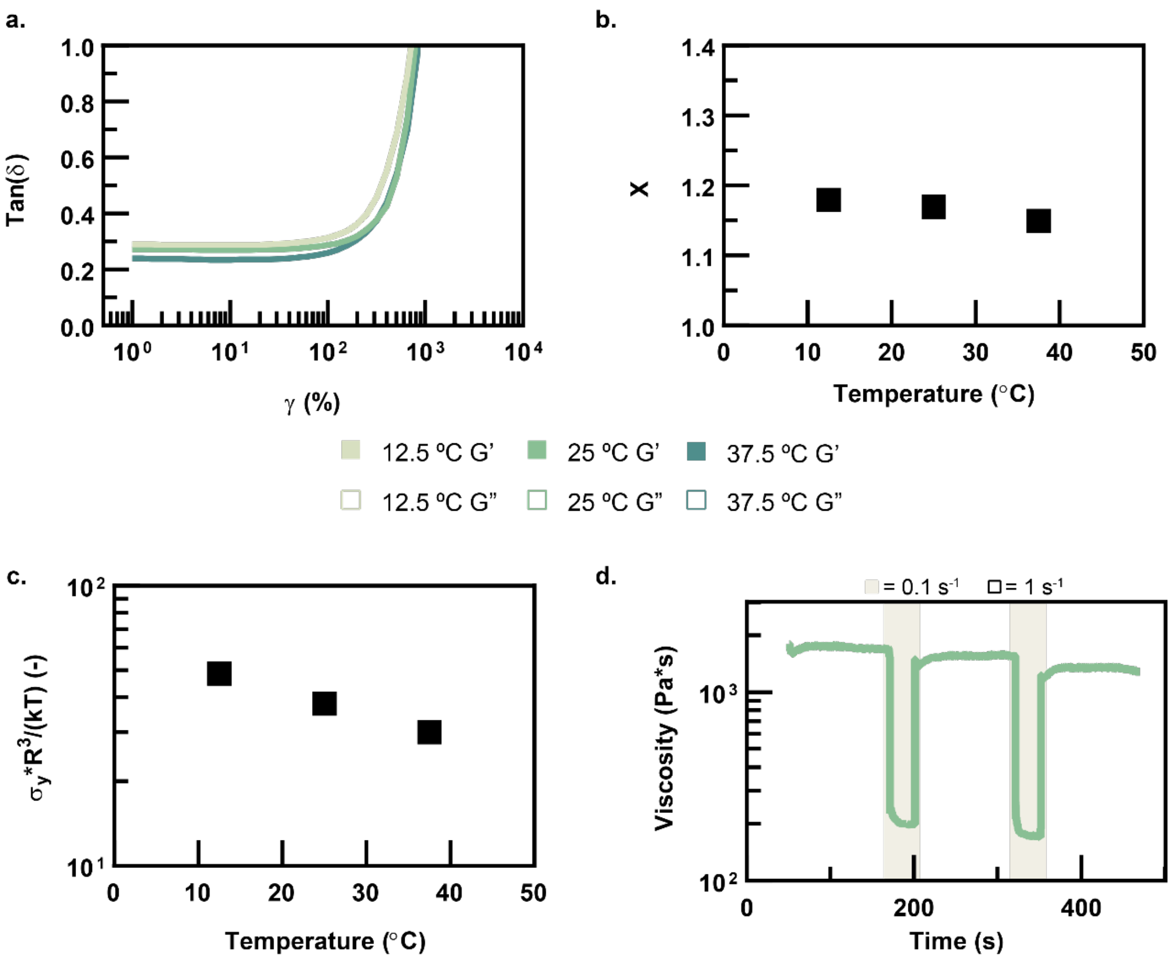

Supplementary Figure 5. Additional analysis of strain sweeps and a step-shear experiment. a, The relative elasticity (tan(δ)) of the amplitude sweeps plotted versus strain, which demonstrates that the PNP gels becomes more elastic at increased temperatures. **b,** The noise temperature, which represents the amount of available energy to locally yield the PNP structure, decreases as temperature increases, indicating the material is more strongly crosslinked at elevated temperatures. **c,** Yield stress values as determined from amplitude sweeps normalized to k_BT/R^3 and plotted versus temperature. Here, the yield stress is defined as the stress at which tan(δ)=1 in the strain sweep. **d,** Step shear experiments alternating between 0.1 and 1 s^{-1} shear rates illustrate the ability for the material to shear-thin and self-heal.

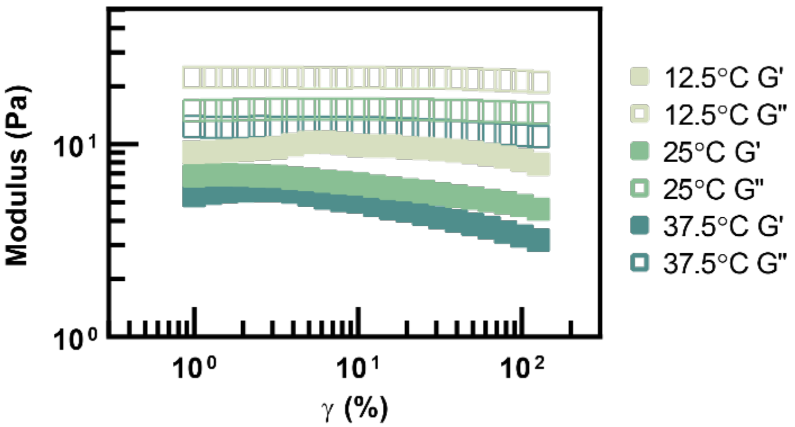

Supplementary Figure 6. Strain sweeps of HPMC-C_{12} solutions. Control experiments illustrate that PSNPs are required to create solid-like materials with tan(δ) values less than 1. Furthermore, HPMC-C_{12} solutions on their own do not exhibit any temperature-induced stiffening or increases in relative elasticity, suggesting the PNP interactions are critical for the temperature behavior observed in the gel formulations.

Supplementary Discussion

In 1946, Green and Tobolsky introduced the molecular theory of polymer networks where the internal crosslinks break and reform at a single rate constant due to thermal fluctuations[4]. This theory was later generalized by Tanaka and Edwards in the early 1990's through a seminal series of papers where they developed several models of the viscoelastic properties of transiently crosslinked networks[51]. Here we briefly discuss the linear response to oscillatory deformations for a model transiently crosslinked network.

This network is assumed to be formed through flexible polymers of uniform molecular weight that have associative groups at either end. Stress induced by small amplitude oscillatory deformations is distributed by elastically active chains, which contributes to the calculated modulus. Furthermore, there is a frequency dependence of the moduli that is directly related to the chain breakage rate, β(T). In the case where there is only one β(T) associated with the network, the storage modulus reduces to a single mode Maxwell model, aptly named the Green-Tobolsky limit:

$$G'(\omega,T) = G_0(T)g(\omega,T) = C\theta(T)\nu_0(T)k_B T \frac{(\omega/\beta(T))^2}{(\omega/\beta(T))^2+1} \qquad (3)$$

where θ(T) is the fraction of bound crosslinks, $v_0(T)$ is the density of crosslinks, C is a junction functionality constant, and the plateau modulus $G_0 = \theta(T)v_0(T)k_BT$. In this paper we take G_0 as the plateau modulus predicted from the affine network model (C=1), which is an overestimate of typical networks and does not account for network loops. Regardless, more complex models such as phantom network theory or real elastic network theory still exhibit a G_0 that is directly proportional to $v_0(T)$ and therefore θ(T),

which possesses crosslink-specific temperature scaling and is why we take the simplest affine network model case[5,6].

The single mode Maxwell model is appropriate because it is the simplest starting model to describe viscoelasticity in associative networks and commonly used to model experimental data of physical networks. Although in many network materials (such as CB[8]/MV/Np-based networks[7], H-bonded networks[8], and metal-ligand networks[9,10]), the terminal relaxation region of the Maxwell model does not fit well, the model is still a reasonable starting point for analysis, especially when there is a only a single G' plateau and single G" inflection point observed in the frequency sweeps. Furthermore, the simplicity of the single mode Maxwell model allows for clean comparison of one fit parameter that enables easy determination of trends. For these reasons, we use the single mode Maxwell to model our frequency-dependent rheometry and stress relaxation data.

As seen in traditional time-temperature superposition, the temperature dependence of β_0 can be introduced through an Arrhenius relation,

$$\beta_0(T) = Aexp\,(-\frac{E_a}{k_B T}) \tag{4}$$

where E_a is the potential barrier for unbinding and A is effectively the attempt rate to overcome the energy barrier[11,12]. In other words, as temperature increases, the chain breakage rate increases, therefore lowering contribution of the frequency-dependent term, g(ω, T), towards the modulus.

Analysis of these equations reveals two results. First, the G_0 contribution to G' results in purely vertical shifts of the frequency-dependent rheology as G_0 is not dependent on frequency. Second, in the Arrhenius construction of β(T), elevated temperatures will only lead to an increase in β(T) for positive E_a values, resulting in a rightward horizontal shift in the frequency-dependent rheology (Supplementary Data Fig. 1). This construction, while physically intuitive, washes out more specific examination of the interacting moieties and precludes the possibility of other temperature-dependent behaviors. These limitations are also apparent in classical derivations of the single-mode Maxwell model, where the number of effective crosslinks (essentially θ(T)) and β(T) are presumed to be Arrhenius[13].

Craig et al. illustrated that the interaction dissociation rate constant (k_d) is in good agreement with β of the same Green-Tobolsky limit equation for G'[9,14]. Using metal-ligand crosslinks, the authors demonstrated that the k_d of the metal-ligand bonds could be substituted for β to model the horizontal shifts in the frequency-dependent rheology. The implication of this finding is that the specific interaction dissociation rate constant of chemical moieties can be used as the crosslink disengagement rate to determine temperature-dependent behavior of bulk mechanical properties[14].

To start, we can represent k_d as

$$k_d(T) = k_a(T)/K_{eq}(T) \qquad (5)$$

where k_a is the Arrhenius-dependent association rate constant and K_{eq} is the equilibrium constant. The Arrhenius dependence of k_a on temperature has been reported for many

systems in the literature to be either small (*e.g.*, due to subtle steric effects) or diffusion limited such that the Arrhenius activation barrier for association at relevant concentrations for network formation is typically less than 10RT[7,15]. Due to the limited range of k_a values observed, the k_d values are often the primary determinant to the large range of K_{eq} values observed[7].

Consequently, consideration of equation 1 with k_d in place of β(T) now affords a temperature dependence rooted in the crosslinking interaction thermodynamics. Specifically, K_{eq} can be represented by the familiar

$$K_{eq} = exp\,(-\frac{\Delta H^{\circ}}{RT} + \frac{\Delta S^{\circ}}{R}) \quad (6)$$

In this equation the temperature dependence lies below the enthalpy term, therefore imposing the temperature dependence of K_{eq} on the sign and magnitude of the enthalpy change.

With these relationships established, we now return to equation 1, where the G' can be analyzed in two parts. First, G_0 is directly proportional to the number of bound crosslinks (θ). If each crosslinking interaction is assumed to be a 1:1 binding reaction (A+B⇌AB), which is a common and appropriate assumption for many systems, θ can be represented by a single site binding model and is related to K_{eq} by

$$\theta = \frac{[B]}{[B]+K_{eq}^{-1}} \quad (7)$$

where [B] is

$$[B] = \frac{-([A]_0-[B]_0+K_{eq}^{-1})^2+\sqrt{([A]_0-[B]_0+K_{eq}^{-1})^2+4\frac{[B]_0}{K_{eq}}}}{2} \quad (8)$$

These relationships provide the dependence of θ on the K_{eq}, which is in turn dictated by the crosslinking enthalpy and entropy changes. Since θ is directly proportional to v_0, equations 6-8 now provide the temperature-dependent behavior of G_0 prescribed by the interaction thermodynamics.

Next, the frequency-dependent term, g(ω, T), can substitute β(T) for k_d to get

$$g(\omega, T) = \frac{(\omega/k_d)^2}{(\omega/k_d)^2+1} \quad (9)$$

$$k_d(T) = k_a(T) exp\,(\frac{\Delta H^{\circ}}{RT} - \frac{\Delta S^{\circ}}{R}) \quad (10)$$

$$k_a(T) = A exp(\frac{E_a}{RT}) \quad (11)$$

These equations illustrate that faster dissociation between dynamic crosslinks results in faster network relaxation times and movement of the rubbery plateau toward higher frequencies (Supplementary Data Fig. 1). Notably, this treatment of g(ω, T) and k_d reveals that endothermic, entropy-driven interactions can result in k_d values that decrease with increasing temperature. As temperature is increased, the non-favorable, endothermic enthalpy term is diminished, leading to smaller k_d values. In turn, this trend is manifested in g(ω, T) whereby the terminal relaxation time of the network is increased and the rubbery plateau is extended to lower frequencies (Supplementary Data Fig. 1). Moreover, the extent to which k_d increases (in the case of a negative ΔH°) or decreases (in the case of a positive ΔH°) depends on the sign and magnitude of ΔS°. This observation suggests that for an entropy-driven interaction where ΔH°~0, changes in temperature do not alter either k_d or g(ω, T).

For the calculations reported in the main text, the k_a values used for each physical interaction were obtained from the literature. The activation energy for the CB[8]/MV/Np hydrogels were chosen to be ~36.5RT (~90 kJ/mol) and the k_a was chosen to be $2.5x10^7$ M^{-1} s^{-1}, which were both reported previously[2,7,16,17]. PNP interaction activation energies were estimated to be ~2.5RT (~6 kJ/mol), which are similar to protein-protein interactions, where steric and conformational barriers lead to larger activation energies[15]. Similarly, the k_a for PNP interactions were estimated to be 10^3 M^{-1} s^{-1}, which is the lower limit of protein-protein interactions exhibiting large steric and conformational barriers. We surmise that polymer-nanoparticle interactions are similar to these protein-protein interactions in that the movement of the polymer chains and high concentrations near the NP surfaces lead to similar steric and conformational barriers. For all systems, interaction $\Delta H°$ and $\Delta S°$ values were assumed to be constant across the tested temperature range (*n.b.*, hydrophobic interactions have been shown to have small temperature-dependent changes in ΔH and ΔS that do not significantly change ΔG, nor the signs of ΔH or ΔS across the tested temperature range[1,18]).

References

1 Rekharsky, M. V. & Inoue, Y. Complexation Thermodynamics of Cyclodextrins. *Chem Rev* **98**, 1875-1918 (1998).

2 Appel, E. A. *et al.* Supramolecular Cross-Linked Networks via Host-Guest Complexation with Cucurbit[8]uril. *Journal of the American Chemical Society* **132**, 14251-14260, doi:10.1021/ja106362w (2010).

3 Fang, Y. P. *et al.* Multiple steps and critical behaviors of the binding of calcium to alginate. *Journal of Physical Chemistry B* **111**, 2456-2462, doi:10.1021/jp0689870 (2007).

4 Green, M. S. & Tobolsky, A. V. A New Approach to the Theory of Relaxing Polymeric Media. *J Chem Phys* **14**, 80-92, doi:Doi 10.1063/1.1724109 (1946).

5 Zhong, M., Wang, R., Kawamoto, K., Olsen, B. D. & Johnson, J. A. Quantifying the impact of molecular defects on polymer network elasticity. *Science* **353**, 1264-1268 (2016).

6 Rubinstein, M. & Colby, R. H. *Polymer physics*. (Oxford University Press, 2003).

7 Appel, E. A. *et al.* Decoupled Associative and Dissociative Processes in Strong yet Highly Dynamic Host-Guest Complexes. *Journal of the American Chemical Society* **139**, 12985-12993, doi:10.1021/jacs.7b04821 (2017).

8 Feldman, K. E., Kade, M. J., Meijer, E. W., Hawker, C. J. & Kramer, E. J. Model Transient Networks from Strongly Hydrogen-Bonded Polymers. *Macromolecules* **42**, 9072-9081, doi:10.1021/ma901668w (2009).

9 Yount, W. C., Loveless, D. M. & Craig, S. L. Small-Molecule Dynamics and Mechanisms Underlying the Macroscopic Mechanical Properties of Coordinatively Cross-Linked Polymer Networks. (2005).

10 Yount, W. C., Loveless, D. M. & Craig, S. L. Strong means slow: dynamic contributions to the bulk mechanical properties of supramolecular networks. *Angew Chem Int Ed Engl* **44**, 2746-2748, doi:10.1002/anie.200500026 (2005).

11 Annable, T., Buscall, R. & Ettelaie, R. Network formation and its consequences for the physical behaviour of associating polymers in solution. *Colloid Surface A* **112**, 97-116, doi:Doi 10.1016/0927-7757(96)03621-7 (1996).

12 Tanaka, F. & Edwards, S. F. Viscoelastic Properties of Physically Cross-Linked Networks .2. Dynamic Mechanical Moduli. *J Non-Newton Fluid* **43**, 273-288, doi:Doi 10.1016/0377-0257(92)80028-V (1992).

13 Tanaka, F. *Polymer Physics: Applications to Molecular Association and Thermoreversible Gelation*. (Cambridge University Press, 2011).

14 Leibler, L., Rubinstein, M. & Colby, R. H. Dynamics of reversible networks. *Macromolecules* **24**, 4701-4707, doi:10.1021/ma00016a034 (1991).

15 Langdon, B. B., Kastantin, M. & Schwartz, D. K. Apparent Activation Energies Associated with Protein Dynamics on Hydrophobic and Hydrophilic Surfaces. *Biophys J* **102**, 2625-2633, doi:10.1016/j.bpj.2012.04.027 (2012).

16 Appel, E. A., Forster, R. A., Koutsioubas, A., Toprakcioglu, C. & Scherman, O. A. Activation energies control the macroscopic properties of physically cross-linked materials. *Angew Chem Int Ed Engl* **53**, 10038-10043, doi:10.1002/anie.201403192 (2014).

17 Tan, C. S. Y. *et al.* Distinguishing relaxation dynamics in transiently crosslinked polymeric networks. *Polym Chem-Uk* **8**, 5336-5343, doi:10.1039/c7py00574a (2017).

18 Ludemann, S., Abseher, R., Schreiber, H. & Steinhauser, O. The temperature-dependence of hydrophobic association in water. Pair versus bulk hydrophobic interactions. *Journal of the American Chemical Society* **119**, 4206-4213, doi:DOI 10.1021/ja953439d (1997).
